# Debilitating Fracture Hampering the Regular Chores of an 80-Year-Old Female and the Post-operative Rehabilitation for the Same: A Case Report

**DOI:** 10.7759/cureus.30245

**Published:** 2022-10-13

**Authors:** Anushka Raipure, Madhu Lakhwani, Pratik Phansopkar

**Affiliations:** 1 Musculoskeletal Physiotherapy, Ravi Nair Physiotherapy College, Datta Meghe Institute of Medical Sciences, Wardha, IND

**Keywords:** post-operative rehabilitation, rehabilitation., physical therapy, intertrochanteric fractures, geriatric population

## Abstract

In the geriatric population, intertrochanteric fractures are exceptionally high because they have osteoporosis. Extracapsular fractures of the proximal section of the femur are known as intertrochanteric fractures. The surgical intervention combined with physiotherapy aids in the achievement of functional objectives. After a fall in the restroom, an X-ray revealed an intertrochanteric fracture of the left hip in an 80-year-old female patient. The concern regarding the surgical intervention was the age and associated co-morbidities. The patient was given physiotherapy for ten weeks after surgical intervention and skeletal traction, which comprised the multidisciplinary approach. The intervention is substantially directed toward balance retraining and improving functional independence. The case report suggests that a structured physiotherapy rehabilitation protocol improved the patient's functional abilities and successful recovery.

## Introduction

The geriatric population has a high incidence of intertrochanteric fracture [1]. Around 80% of hip fractures occur in women, and the average age for hip fractures is 80 years. Many hip fractures are associated with falls, but other risk factors include reduced bone mineral density and decreased activity levels [2]. There is the deterioration of bone density with increasing age, commonly known as osteoporosis. Osteoporosis ultimately results in increased risks of falls and related injuries [3]. As the expectancy of life in the geriatric population has gradually increased, there is a substantial rise in the prevalence of hip fractures. Such injuries will likely grow in the coming decade [4].

Extracapsular fractures in the proximal section of the femur, commonly between the greater and lesser trochanters, are known as intertrochanteric fractures. The thick trabecular bone composes the intertrochanteric region of the femur. The gluteus medius, gluteus minimus, obturator internus, and piriformis all insert over the greater trochanter of the femur, although the vastus lateralis originates there [5].

## Case presentation

An 80-year-old female was brought to casualty with pain and swelling over her right hip. The patient gave a history of slip and fall in the bathroom at home on February 2, 2021, sustaining an injury to the right hip. The pain felt was sudden in onset and gradually increasing in nature. There was no history of head injury, loss of consciousness, and ENT bleeding. The patient is an investigated bronchial asthma patient on medications. There was no significant past or family history. The patient was on skeletal traction for 13 days; then, on the 14th day, she was operated. Table 1 shows the order of the events.

**Table 1:** Shows the timeline of the events.

Event	Date
Date of fall	February 2, 2021
Date of admission	February 2, 2021
Date of Operation	February 15, 2021
Date of physiotherapy rehabilitation	February 17, 2021

Clinical findings

Before the examination, the therapist took the consent of the patient. The patient was examined in a supine position with both anterior superior iliac spines (ASISs) at the same level. On inspection of the right hip, the overlying skin appeared normal. No noticeable swelling was present. The patient's right lower extremity was externally rotated and abducted. Also, the right greater trochanter seemed to be broad and at a higher level. The right lower limb appears shorter. On palpation, local temperature was normal. There was a presence of bony tenderness over the right greater trochanter. There was a 1.5 cm shortening of the right lower limb. 

Diagnostic assessment

**Figure 1:**
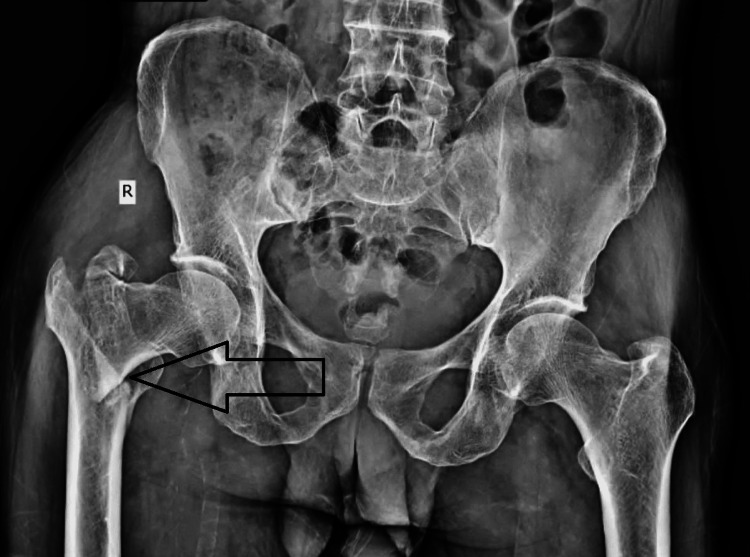
Pre-operative radiograph of the pelvis and both hips showed an intertrochanteric fracture of the right femur.

A pre-operative radiograph of the pelvis and both hips showed an intertrochanteric fracture of the right femur with subtrochanteric extension (Figure 1).

**Figure 2:**
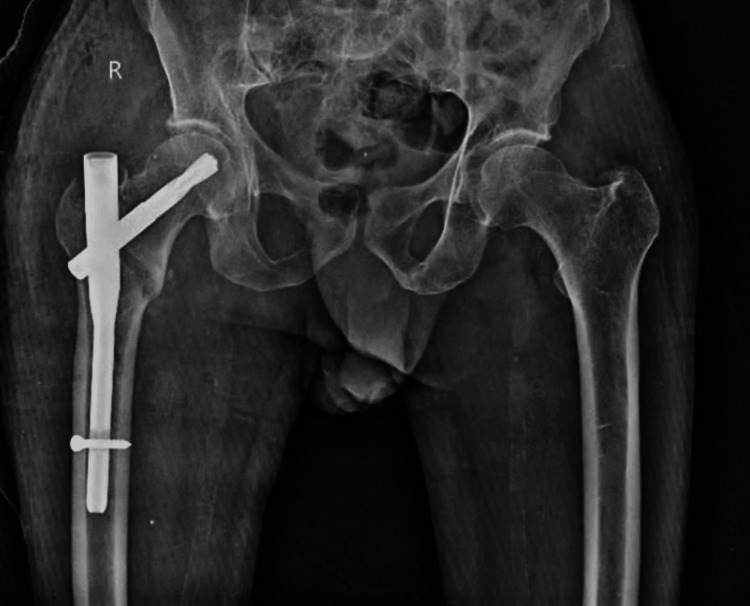
Post-operative x-ray of right femur.

Figure 2 shows a post-operative X-ray.

The pre-rehabilitation assessment included the assessment of the range of motion and manual muscle testing. Findings of the pre-operative evaluation are shown in Table 2 and Table 3, respectively.

**Table 2:** Range of motion assessment pre- and post-rehabilitation.

Joint	Pre-rehabilitation			Post-rehabilitation		
		Active	Passive		Active	Passive
Hip flexion		Unable to perform	Unable to perform		0-90	0-100
Extension (hyper)					0-18	0-22
Abduction					0-40	0-45
Adduction					0-25	0-28
Knee flexion					0-130	0-135
Extension					130-0	135-0

**Table 3:** Manual muscle testing assessment pre- and post-rehabilitation.

Joint		Pre-rehabilitation	Post-rehabilitation
Hip	Abductors	Unable to perform	4+/5
	Adductors		4/5
	Flexors		4/5
	Extensors		4/5
	Internal rotators		3+/5
	External rotators		4+/5
Knee	Extensors		4+/5
	Flexors		4/5

Pain assessment on the Numeric Pain Rating Scale (NPRS) was 7/10 on rest and 9/10 on movement. The lower limb functional test score was 28/80. Timed Up and Go test finding pre-rehabilitation with a walker was 26.3 seconds. The fall risk assessment score pre-rehabilitation was 18.

Physiotherapy intervention

The therapist prepared a specific protocol according to the patient's condition.

Phase 1 (Weeks 1- 6)

**Figure 3:**
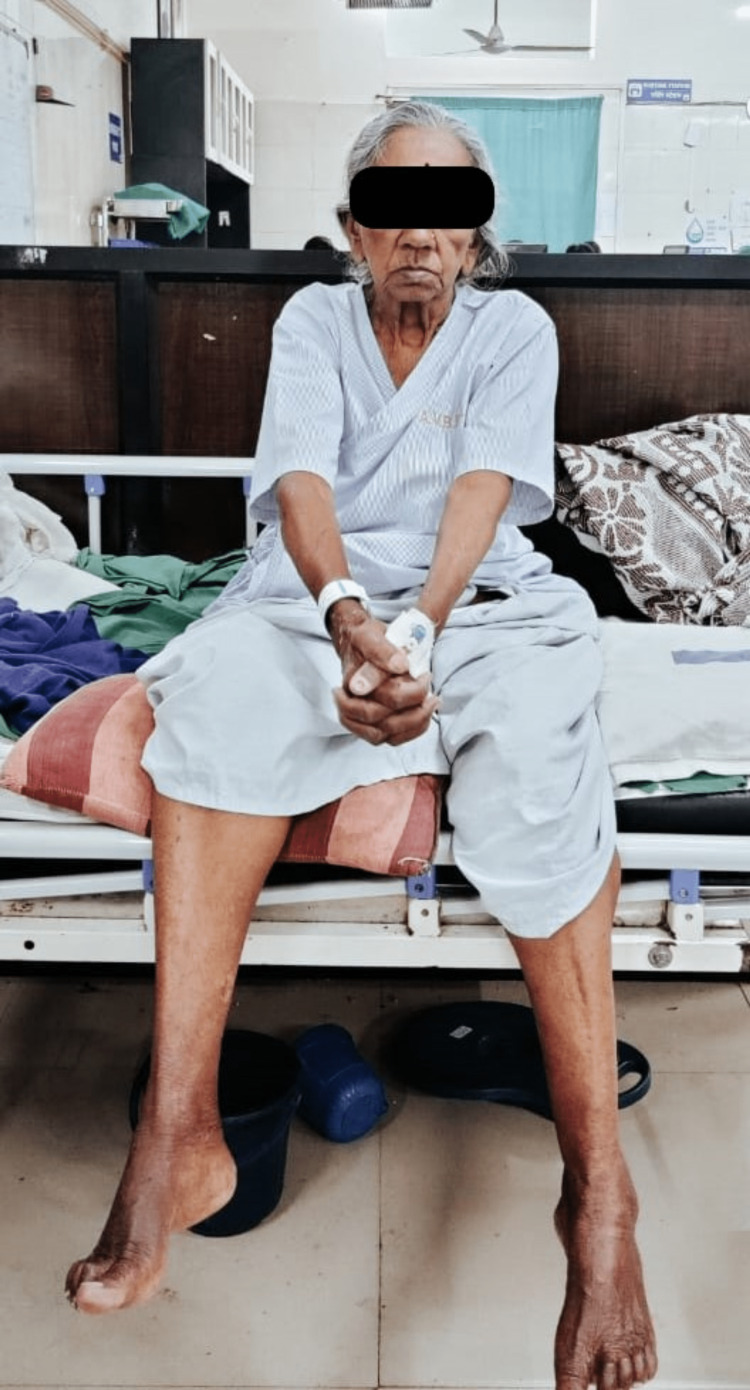
Shows the patient doing breathing exercises.

The therapist started physical therapy as a gentle range of motion exercise and initiated lower extremity isometric exercises. The initial focus of the treatment was to avoid the development of knee flexion contracture. Therefore attaining early knee extension was the primary focus of the treatment. Early knee extension was achieved by lower extremity exercises like static hamstring stretching and quadriceps stretching. Along with these exercises, heel propping exercises were administered for 10 minutes, facilitating posterior stretching of the knee. As the patient also had a history of asthma, the therapist helped with pursed-lip breathing and thoracic expansion exercises (Figure 3) to prevent tachypnea and facilitate effective breathing.

Along with exercises, the patient underwent electrotherapy treatment. The therapist stimulated the quadriceps to regain voluntary control. The initial focus of the strengthening exercises was to regain active control of knee extensors and hip abductors. Strengthening began in non-weight-bearing positions, such as open kinetic chain workouts. Keeping a strong quadriceps contraction while performing these exercises with the knee fully extended was important. One of the earliest attempts to engage the quadriceps muscle and lessen the likelihood of knee extensor lag, which is frequent in adults with intertrochanteric fractures, was made in this exercise. Hip abduction strength was chosen as a target because patients with intertrochanteric fractures frequently exhibit altered gait. The focus was on the exercises of the soleus and gastrocnemius muscles. The patient made a progressive switch from non-weight-bearing to weight-bearing activities following the patient's comfort and tolerance level. The patient performed balance and proprioception exercises when the weight-bearing exercises were initiated. After beginning easy weight-bearing workouts, the lower extremity's closed kinetic strengthening protocol was initiated.

Additionally, she learned toed raises to prepare her for successful weight bearing. The goal of the first gait training exercises was to normalize the temporal or spatial gait variables and encourage knee flexion during the swing portion of the gait cycle. Before progressing to phase 2 of rehabilitation, we ensured the patient could bear 50% of her body weight with an assistive device. The patient performed fair quadriceps contraction and also demonstrated fair hip abduction by the end of phase 1.

Phase 2 (Weeks 6-8)

The patient continued with phase 1 exercises. Weight-bearing was now encouraged without assistive devices. The range of motion exercises administered during phase 1 continued in phase 2. General lower extremity stretching focused on the gastrocnemius, soleus, and hamstrings was continued as tolerated by the patient. Progression of range of motion was of focus in phase 2 as well. Maintaining full knee extension and gradual progression to knee flexion activities were carried out. Here in phase 2, there was a noticeable progression in weight-bearing efficiency. In phase 2, closed chain kinetic strengthening activities showed noticeable improvement. The gait training activities, balance, and proprioception activities also progressed. Sidestepping was administered. To facilitate effective breathing, the patient continued to do pursed lip breathing.

Before progressing the patient to phase 3 of rehabilitation, the patient could bear her total body weight without an assistive device. Manual muscle testing revealed quadriceps strength of 4+/5. On a manual muscle test, the hip abductor strength was 4/5.

Phase 3 (Weeks 8-10)

Phase 3 was focused on strength development, normalizing gait, and returning to activities of daily living. The patient continued range of motion exercises as per phase 2. Stretching exercises initiated in phase 2 were continued and progressed as indicated and tolerated by the patient-gradual progression in strengthening exercises by gradually increasing weights as per the patient's tolerance. Hip abduction strengthening also progressed. Balance and proprioception activities were also advanced as tolerated since the patient was full weight-bearing. The patient continued with the breathing exercises.

After phase 3, the therapist noted the following outcomes. The patient presented with standard gait patterns. Manual muscle testing revealed quadriceps strength of 4/5. On a manual muscle test, the hip abductor's power was 4+/5.

Followup and outcomes

**Figure 4:**
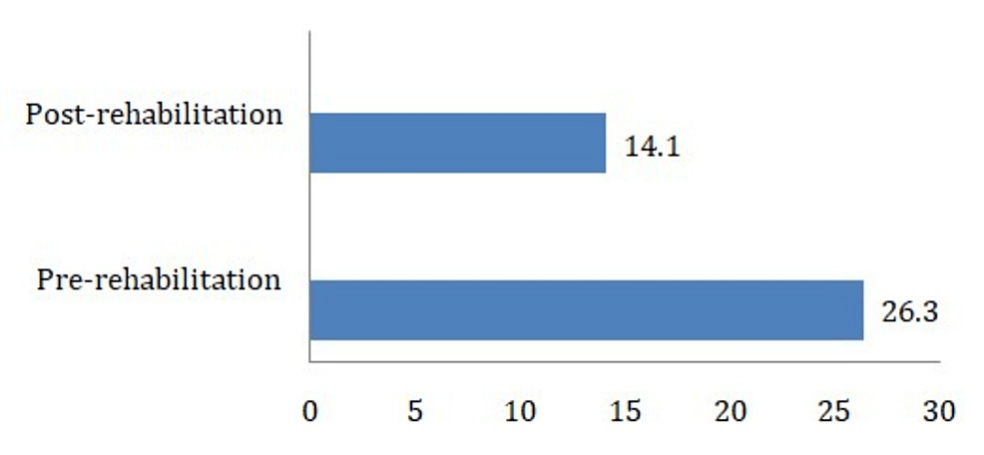
Shows a comparison of the Timed Up and Go test between pre- and post-rehabilitation.

The patient demonstrated the following improvements following 10 weeks of physical therapy rehabilitation. There was a significant improvement in range of motion and muscle strength. Pain assessment findings for post-rehabilitation pain on NPRS was 1/10 on rest and on movement 2/10. Lower Limb functional test gave a pre-rehabilitation score of 28/80 and the post-rehabilitation score was 56/80. Timed Up and Go test findings were as follows: pre-rehabilitation: with walker- 26.3 seconds (indicates balance problems and gait difficulties), and post-rehabilitation: 14.1 seconds under supervision (indicates a slight fall risk but with enhanced mobility) (Figure 4).

**Figure 5:**
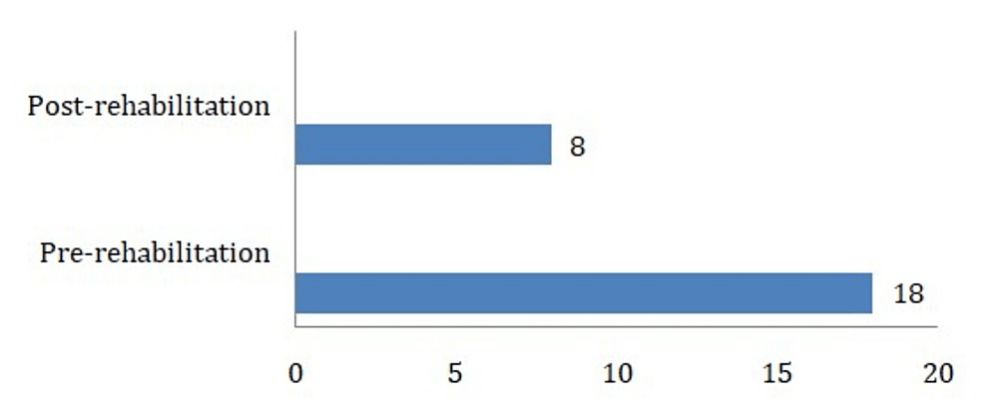
Shows the comparison between the pre- and post-rehabilitation scores of fall risk assessment.

Fall risk assessment score pre-rehabilitation was 18, indicating high fall risk; it improved post-rehabilitation to 8, indicating moderate fall risk (Figure 5).

## Discussion

Hip fractures are a contentious issue all around the world. Geriatric hip fractures are becoming a common concern among the world's aging population. Most hip fractures are induced by osteoporosis and fall in everyday pursuits, with men outnumbered by women by 1:4. Between 40% and 60% of older people will get a femoral neck fracture or trochanteric fracture. Femoral neck fractures are less frequent in the elderly than trochanteric fractures [1].

This study reports the triumphant conventional treatment of intertrochanteric fracture, which was stable and non-displaced. In the geriatric population, there is an emphasis on conservative management as there is a risk of potential complications. Therefore, conservative management is a fundamental treatment in geriatric patients. Several studies suggest prompt reduction and internal fixation improve patient comfort, optimize nursing care, encourage early mobility, and shorten hospital stays in senior patients [6]. Studies have demonstrated the efficacy of nonsurgical therapy for intertrochanteric femur fractures in older persons. With 170 patients over 50 who had intertrochanteric fractures, Horn and Wang discussed their traction treatment. The patients were given 6-8 weeks of traction along with knee movements and full weight-bearing after 12 weeks. All patients who previously ambulated could do so again, and few complications were recorded [7].

Mascot and Herickhoff discuss an instance of a non-displaced intertrochanteric femur fracture which they could successfully treat with conservative therapy and limited weight-bearing. While there have been reports of effective conventional therapy for this injury, operational treatment is still the gold standard for many individuals with comparable injuries, such as those older than 40 and those with unstable intertrochanteric femur fractures [8].

According to a retrospective cohort research by Chelbeck et al., geriatric patients who received conservative care experienced a considerably more extended hospital stay than those who received surgical treatment. The conventional treatment included early mobilization and restricted weight bearing according to the patient's comfort [9]. Bong et al. studied 150 unstable intertrochanteric fractures treated with traction and surgery. The authors discovered that younger and active individuals with good healthcare services reacted best to conservative therapy, but all three groups had similar functional results. The conventional treatment consisted of skeletal traction with a tibial pin for 12 weeks along with lower limb strengthening [10].

The analysis by Lima et al. suggests a home-based workout regimen that might help elderly patients who have trouble using public transit or don't have a caretaker to accompany them to sessions overcome some of the obstacles to rehabilitation. The program consisted of balance and lower limb training. The lower limb strengthening protocol is gradually progressing with an emphasis on hip abductors, knee extensors, and ankle dorsiflexors. The therapist should incorporate it into the rehabilitation protocol because it benefits the patients [11]. According to Stahl and Westerdahl, people over 80 may benefit from more intensive physiotherapy after a hip fracture is surgically repaired. Intensive physiotherapy can be given in terms of early mobilization, strengthening of lower limbs, bedside exercises, and breathing exercises [12].

## Conclusions

Intertrochanteric fractures are treated surgically and afterwards with physical therapy. In this case, the patient falls into the geriatric population. With significant improvements in both physical and mental well-being, the post-fracture rehabilitation programme is successful. The case study illustrates a thorough method for treating patients post-fracture surgery. The patient achieved most of the essential objectives following a rehabilitation protocol. Enhanced muscle strength, increased hip range of motion, improved cognition, pain reduction, and improvements in the patient's gait and daily activities were clinically significant after 12 weeks of concentrated physical therapy. According to this study, the patient's functional goals were improved through early physiotherapy rehabilitation.
